# Molecular Views into the Synthesis and Activation of Flavocytochrome *b*_558_ in Phagocytic Cells—Focus on the Role of EROS

**DOI:** 10.3390/antiox15060724

**Published:** 2026-06-06

**Authors:** Perrine Rochas, Maria Val-Pevida, Sylvain Beaumel, Isabelle Petit-Härtlein, Caroline Plazy, Franck Fieschi, Marie José Stasia

**Affiliations:** 1 Institut de Biologie Structurale, UMR5075, CNRS, CEA, Université Grenoble Alpes, 38042 Grenoble, France; perrine.rochas@ibs.fr (P.R.); maria.val-pevida@ibs.fr (M.V.-P.);; 2 CDiReC, Pôle Biologie, Centre Hospitalier Universitaire Grenoble Alpes, 38043 Grenoble, France; 3 Institut Universitaire de France (IUF), 75231 Paris, France

**Keywords:** NOX2, EROS, NADPH oxidase, flavocytochrome *b*_558_, reactive oxygen species

## Abstract

Recent advances in the structural characterization of the phagocyte NADPH oxidase coupled with the description of its chaperone EROS for Essential for Reactive Oxygen Species have led to a better understanding of its function and activation in phagocytic cells. This review examines the role of EROS chaperone in flavocytochrome *b*_558_ biosynthesis and function and in physiological and pathological conditions. Based on experimental data and structural insights, we synthesize knowledge from former work on flavocytochrome *b*_558_ synthesis and structure combined with recent advances on the specific role of EROS chaperone on the potential control of Reactive Oxygen Species (ROS) production by c flavocytochrome *b*_558_. We particularly emphasize its role in the pathological context of Chronic Granulomatous Disease (CGD), with already described EROS mutations (known as CGD5), as well as rare X91^minus^-CGD (or X91^−^-CGD) cases characterized by low flavocytochrome *b*_558_ expression in phagocytes that could be due to a lack of interaction with EROS. Future works should address in more detail how EROS binding and release from flavocytochrome *b*_558_ is regulated, and whether the inhibitory effect on ROS production that was observed in EROS overexpression studies is relevant in a more physiological context.

## 1. Introduction

Phagocytes, mainly monocytes, macrophages and neutrophils, are crucial cells to protect the body from microbial infections . Thus, the physiological role of phagocytes is to ingest foreign bodies, including microbes and cellular debris, for their elimination. Although phagocytes have a broad panel of microbicidal mechanisms, including antimicrobial peptides and lytic enzymes secretion, reactive oxygen species (ROS) produced by the activated NADPH oxidase complex remains the main mechanism responsible for the elimination of most invading pathogens. This enzymatic complex is composed of at least five specific subunits—the membrane proteins NOX2 (also called gp91*^phox^*) and p22*^phox^* forming the flavocytochrome *b*_558_ heterodimer and the cytosolic factors p47*^phox^*, p67*^phox^* and p40*^phox^*. In addition to these, a sixth essential partner required for optimal activity is a small G protein that can be either Rac1 or Rac2. In its resting state, this complex is dissociated and inactive. The specific recognition of opsonized pathogens by their specific receptors on phagocytic cells triggers a cascade of phosphorylation, particularly at the level of p47*^phox^*, which induces the assembly of the NADPH oxidase complex subunits for its activation in order to catalyze the production of superoxide anions O_2_^−^ within the newly formed phagosome containing the pathogen [1]. Superoxides are precursor molecules for the generation of a variety of microbicidal oxidants such as H_2_O_2_, OH, HOCl.

The fundamental importance of this defense process is illustrated by the impact of its dysfunction in chronic granulomatosis disease (CGD). CGD is a rare inherited syndrome (frequency of approximately 1/200,000), characterized by a defect in ROS production by the NADPH oxidase complex, leading to severe and recurrent infections in childhood and chronic inflammatory granulomas. Indeed, patients with CGD are at a markedly increased risk of developing invasive bacterial and fungal infections affecting virtually any organ system, with a particular predilection for the lymph nodes, liver, and lungs. The most frequently isolated pathogens are *Staphylococcus aureus* and *Aspergillus species*. Recurrent cutaneous infections caused by *S. aureus* are also commonly observed. In addition to infectious complications, patients often develop autoinflammatory manifestations, notably inflammatory bowel disease (IBD), which can be challenging to control with conventional immunosuppressive therapies. Consequently, many patients remain dependent on long-term corticosteroid treatment [2]. The diagnosis of CGD is based on the assessment of ROS production by the phagocyte NADPH oxidase complex. ROS generation is typically measured in peripheral blood phagocytes following in vitro stimulation with phorbol 12-myristate 13-acetate (PMA) or particulate agonists such as opsonized zymosan using flow cytometry-based dihydrorhodamine (DHR) assays [3]. Conservative management requires strict adherence to hygienic measures and lifelong antimicrobial prophylaxis. Trimethoprim-sulfamethoxazole (cotrimoxazole) and triazole antifungal agents constitute the cornerstone of prophylactic therapy. Owing to continuous improvements in diagnostic approaches, infection prevention strategies, and supportive care, life expectancy in conservatively treated patients has substantially improved and may increasingly exceed the previously reported range of 30–40 years [4]. Nevertheless, persistent or recurrent severe infections and refractory autoinflammatory complications represent major indications for curative cell-based therapies, including allogeneic hematopoietic stem cell transplantation (HSCT) from a healthy donor and autologous gene therapy using genetically corrected hematopoietic stem cells [5,6,7]. In patients without active infection or uncontrolled inflammation, HSCT provides an excellent probability of long-term survival and disease cure. When an HLA-matched donor is unavailable, transplantation from a haploidentical parental donor has become a feasible and effective option for most infants and children. In the absence of a suitable donor, gene therapy may constitute an alternative therapeutic strategy. Gene therapy is also being considered for patients with a high disease burden, as the conditioning regimens employed are generally less intensive than those required for allogeneic HSCT. However, data from recent clinical trials indicate that mortality rates remain higher following gene therapy than after HSCT [8,9]. This difference is likely attributable, at least in part, to the more severe clinical status of patients enrolled in gene therapy studies, although a higher incidence of graft failure or loss of corrected cells may also contribute to inferior outcomes.

The classical genetic forms of CGD are caused by mutations in X-linked recessive mutations in *CYBB* (X-CGD), or autosomal recessive mutations (AR-CGD) in *CYBA*, *NCF1*, and *NCF2* encoding NOX2, p22*^phox^*, p47*^phox^*, and p67*^phox^* subunits. Beside these classical CGDs, the clinical table of p40*^phox^* deficient patients caused by mutations in *NCF4* underlies a distinctive condition, resembling a mild, atypical form of CGD. Only 24 p40*^phox^*-deficient patients with 10 different mutations were described up to now [10]. Nearly at the same time, two teams described for the first time homozygous loss-of-function human mutations in the *CYBC1* (for cytochrome *b*_558_ chaperone 1) gene encoding EROS protein leading to a new form of AR-CGD5 [11,12]. The clinical presentation of EROS-deficient patients looks like AR-CGD often with auto-inflammatory and chronic inflammation manifestations beside infections. The essential role of EROS for the phagocyte respiratory burst was highlighted one year before in a mouse deficient in a previously uncharacterized gene *bc017643* highly susceptible to *Salmonella* and *Listeria* infection [13]. Interestingly, EROS has a plant ortholog Ycf4 which is necessary for the expression of photosystem I subunits in chloroplasts [14]. David Thomas’ team demonstrated that EROS is also a chaperone necessary for stable expression of the NOX2 and p22*^phox^* membrane subunits of the flavocytochrome *b*_558_ heterodimer [15].

In this review, we will look back at the work that led to the elucidation of the flavocytochrome *b*_558_ synthesis and discuss it in the light of its new partner EROS. Thanks to the latest knowledge of the structure of the EROS/NADPH oxidase complex, we will analyze the effect of some mutations of NOX2 in regions at the interface with EROS leading to rare X91^−^-CGD variants affecting cytochrome *b*_558_ expression and/or enzymatic activity.

## 2. The Phagocytic Flavocytochrome *b*_558_

### 2.1. The Discovery of the Flavocytochrome b_558_ as the NADPH Oxidase in Neutrophils Thanks to CGD

The father of the flavocytochrome *b*_558_ is undoubtedly Pr. Anthony W. Segal from the University College London, UK. It all began when, as a junior doctor, he became interested in the biochemical reaction of the nitroblue tetrazolium (NBT) reduction in neutrophils for the diagnosis of pyogenic infections [16]. However, this test was not discriminating for this diagnosis because of false positives due to the presence of fibrinogen and heparin in the blood. At the same time, Robert Good’s team in the USA discovered a new syndrome affecting children, a fatal granulomatosis, probably of genetic nature and characterized by the absence of respiratory burst of neutrophils [17,18,19]. Then, Baehner and Nathan demonstrated that the neutrophils of two children suffering from this chronic granulomatous disease failed to reduce NBT during phagocytosis [20]. From this moment, various teams around the world set out to purify and identify the ROS-producing NAD(P)H oxidase that was absent in CGD neutrophils.

To make a long story short, it was Antony W. Segal in collaboration with Owen Jones from the University of Bristol (the Brian Chapell team), that identified a new low-potential cytochrome *b*_-245_ (with a midpoint potential of −245 mV) as an essential component of the microbicidal oxidase system in neutrophils [21]. However, we should not forget to mention two Japanese teams who, ten years earlier, had identified the spectrum of a cytochrome *b* in horse and rabbit neutrophils, but no link with the ROS-producing NADPH oxidase had been established [22,23]. Indeed, this cytochrome (with a characteristic peak at 558 nm in his reduced-minus oxidized spectrum), was absent in neutrophils of X91^0^-CGD patient and in reduced concentration in two mothers known to be carriers of this disease. Thus, this cytochrome was a strong candidate to be the NADPH oxidase itself [24]. However, Borregaard et al. in 1979, published CGD cases, where one male patient with an X-linked transmission had a normal amount of cytochrome *b*_558_ [25]. In fact, it was the first description of a X91^plus^-CGD variant named X91^+^-CGD characterized by a normal expression of an inactive cytochrome *b*_558_ [26,27]. The genetic base of X-linked CGD was evidenced when in 1986, Royer-Pokora et al. cloned the gene for X-linked CGD by relying on its chromosomal position [28]. The corresponding protein sequence matched perfectly with the nucleotide sequence of the purified heavy chain (also named β-chain) of cytochrome *b*_558_ from neutrophils [29] and was identified by Western blot with specific antibodies [30]. NOX2 is encoded by the *CYBB* gene (OMIM number 306400) contains 13 exons which spans 33.5 kb and is located at position Xp21.1-p11.4 in the short arm of the X-chromosome.

In the 80s, the majority of teams working on the subject thought that cytochrome *b* was part of a complex with an NADPH-dependent flavo-oxidase responsible for ROS production in stimulated neutrophils. However, the characterization of cytochrome *b*_558_ continued and Antony W. Segal and coworkers demonstrated that partially purified cytochrome *b*_558_ from human neutrophils identified two subunits: one around 76–92 kDa and a second one at 23 kDa [31]. Nearly at the same time, the team of Algirdas J. Jesaitis in the US purified the cytochrome *b*_558_ as two components of 91 (the heavy chain, or the b-chain; or NOX2, or gp91*^phox^*) and 22 kDa (the light chain or the α-chain, or p22*^phox^*), the highest subunit being N-glycosylated [32]. Both teams revealed that this cytochrome contains hemes but the positioning of them remained unknown [33,34]. First of all, it was the small subunit that was identified as carrying the hemes [34,35]. The study of a X91^+^-CGD variant with a Arg54Ser mutation was decisive to understand the strange low midpoint potential of cytochrome *b*_558_. Indeed, the cytochrome *b*_558_ spectrum of this mutant showed a slight shift in the differential spectrum that led to reanalyzing its potential mid-point [36]. The presence of two nonidentical hemes with different midpoint potentials was shown in the neutrophils of this X91^+^-CGD patient. Reanalysis of redox titrations of normal cytochrome *b*_558_ revealed the presence of two hemes with closely spaced midpoint potentials which merged to give the −245mV value. Then identity of the heme-binding histidines were predicted *via* the study of the FRE1 reductase of the yeast *Saccharomyces cerevisiae*, homologous to NOX2. By directed mutagenesis, four histidines in FRE1 analogs to His101, His115, His209 and His222 in NOX2, were identified as the heme-binding residues [37]. The human His101Tyr X91^−^-CGD mutation revealed a defect in NADPH oxidase activity with a defective differential spectrum of cytochrome *b*_558_ and in NOX2 expression too. These results demonstrated that His101 of NOX2 was one of the ligands for the heme groups of cytochrome *b*_558_ [38]. A few years later, Mary C. Dinauer and co-workers confirmed the importance of these His on the binding of both hemes in NOX2 for the maturation of cytochrome *b*_558_ [39]. Finally, glycosylation sites on the large cytochrome *b* subunit NOX2 were revealed. This N-glycosylation appeared on residues Asn131, Asn148 and Asn239 [40]. The importance of the glycosylation process of cytochrome *b*_558_ will be discussed in the next section.

**Figure 1:**
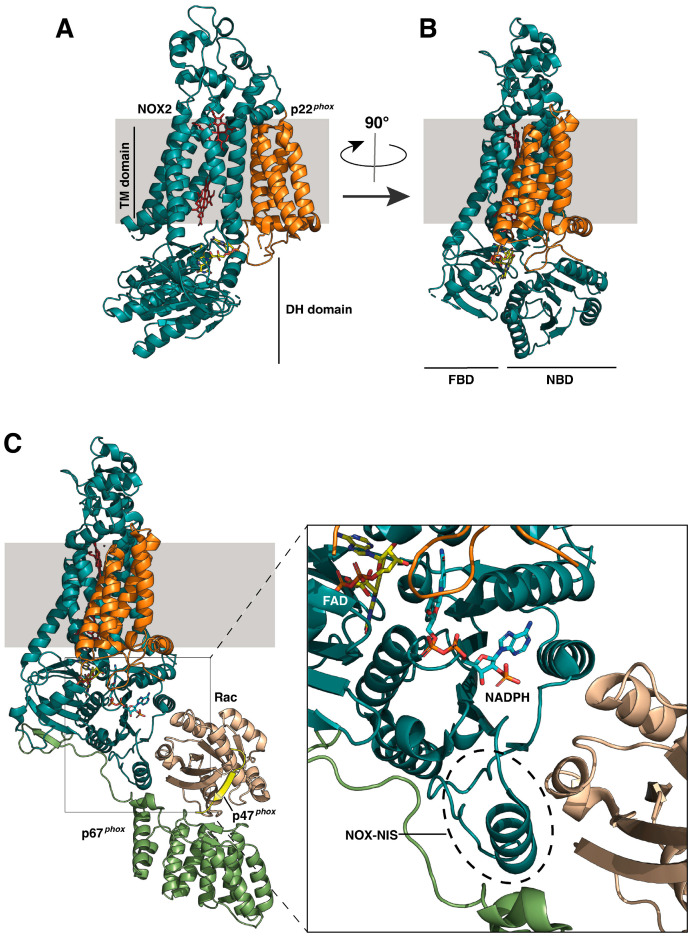
The structure of the human NOX2/p22*^phox^* complex in the resting and activated states: (**A**) and (**B**) Structure of the NOX2/p22*^phox^* complex in the resting state (PDB ID: 8gz3) in cartoon representation viewed parallel to the membrane plane (**A**) or rotated by 90° clockwise presenting p22*^phox^* on the front (**B**), where the Flavin-Binding sub-Domain (FBD) and NADPH-binding sub-Domain (NBD) can be distinguished. NOX2 is composed of six transmembrane helices: the heme-binding ferric oxidoreductase transmembrane domain (TMD) and the dehydrogenase domain (DHD) composed of the FBD and NBD. The approximate boundaries of the phospholipid bilayer are indicated as thick gray lines. NOX2 and p22*^phox^* are colored in deep teal blue and orange colors respectively. Hemes are colored in red and FAD is represented in sticks using CPK colors, but yellow for carbon atoms; (**C**) Structure of the NOX2/p22*^phox^* complex in the activated state (PDB ID: 8wej) representing p22*^phox^* in the front with the assembly of the corresponding cytosolic factors required for the activation of the complex: p67*^phox^*, Rac and p47*^phox^*. Structurally characterized parts of NOX2, p22*^phox^*, p67*^phox^*, Rac and p47*^phox^* are colored in deep teal blue, orange, green, pale brown and yellow respectively. The binding of the cytosolic factors promotes rotation and contraction of the DH domain allowing its docking onto the bottom of the TMD enabling the electron transfer. NADPH is represented in sticks using CPK colors, but blue for carbon atoms. Close-up view highlighting the appearance of the NOX-NIS structural element in the activated NOX2-DH domain upon complex-formation with p67*^phox^* and Rac. NOX-NIS is disordered and not visible in the structure in the resting state (**A**,**B**). Figures were made using PyMOL molecular graphic system v3.1.6.1 and assembled using Adobe Illustrator 30.2.1.

Perhaps the most striking advance in the beginning of 90s in this field was the evidence provided by the structural analogy of the C-terminal dehydrogenase (DH) domain of the cytochrome *b*_558_ with members of the ferredoxin reductase family, demonstrating that this cytochrome contained an NADPH- and a FAD-binding sites as an NADPH oxidase [41,42,43]. Shortly afterwards, a structural model for the nucleotide-binding domain or dehydrogenase domain (DH) of the flavocytochrome *b*_558_ beta-chain was proposed [44]. It allowed the prediction of two subdomains, within this ferredoxin reductase-like domain, as the Flavin-Binding sub-Domain (also called FBD) and the NADPH-binding sub-Domain (NBD). This model has been very useful for two decades in exploring the functioning of the NOX2 protein. It is also the first model to have highlighted a unique insertion sequence of 20 residues in the NBD part of the dehydrogenase domain of NOX enzymes but absent in other classical ferredoxin reductases. Already at that time, a regulatory role in the activation of the enzyme was proposed for this insertion sequence. Through the years, the identification of many X91^+^-CGD mutations in this insertion supported the central role of this sequence and notably its role in the correct assembly with p67*^phox^*, the activator subunit of the NADPH oxidase complex [45]. More recently, its phosphorylation by ATM has been pointed out as a way to down-regulate the activity of NOX2 [46]. This structural element which we have named NOX-NIS (standing for “NOX-NADPH-domain Insertion-Sequence”), has had its regulatory function confirmed by the recent structural determination of the activated state of NOX2, where the NOX-NIS element serves as the central docking site for both p67*^phox^* and Rac2, thereby triggering the conformational change in the DH domain required for activation (Figure 1C) [47].

The identification of the small subunit of flavocytochrome *b*_558_ (p22*^phox^* or the a-chain) was done in parallel. First, it was identified during the flavocytochrome *b*_558_ purification (see above). The primary amino acid sequence of p22*^phox^* was determined from cloned cDNA but the topologic organization of the p22*^phox^* and NOX2 subunits was not known at that time [48]. The absence of p22*^phox^* in the neutrophils from X-CGD patients highlighted the importance of the presence of both the NOX2 and p22*^phox^* subunits for the flavocytochrome *b*_558_ expression [49]. This point will be developed in the next section. Then, Mary C. Dinauer and coworkers characterized the *CYBA* gene structure and location and revealed a new form of autosomal recessive CGD (AR-CGD) caused by mutations in the *CYBA* gene encoding p22*^phox^* [50]. P22*^phox^* is a ubiquitous protein encoded by the *CYBA* gene (OMIM number 233690) located on the long arm of chromosome 16 at position q24, containing six exons and spanning 8.5 kb (OMIM 608508). Numerous polymorphisms have been described associated with pathologies (for reviews see [51,52]). In addition, p22*^phox^* also participate in NADPH oxidase activation. Upon PMA activation, p22*^phox^* is phosphorylated on Thr147 enhancing the NADPH oxidase activity by promoting p47*^phox^* binding [46,53]. This Thr is closed to the polyprolin rich (PPR)-domain (Lys149–Glu162) first identified as an interacting sequence with p47*^phox^* during NADPH oxidase activation [54,55]. In parallel with the phosphorylation of p22*^phox^*, further cumulative phosphorylation on p47*^phox^* itself leads to a significant structural reorganization of this protein, which transitions from a self-inhibited state to an unlocked state, thereby enabling it to bind to p22*^phox^* [56,57,58]. This structural transition thus enables the translocation of p47*^phox^* to the membrane, thereby transporting p67*^phox^*, its partner, to NOX2 for enzymatic activation. The importance of this p22*^phox^*/p47*^phox^* interaction for NADPH oxidase activity was evidenced by the study of an AR22^+^-CGD case caused by a Pro156Gln substitution in p22*^phox^*, leading to defective translocation of p47*^phox^* [59]. Indeed, the consensus sequence PxxP within this domain is able to interact with the tandem of SRC homology 3 (SH3) domains of p47*^phox^*, but also, similarly, with its homolog, the cytosolic organizer NOXO1 [60,61,62]. A crystal structure confirmed this interaction and highlighted important residues such as Pro152, Pro156 and Arg158 [63,64,65].

The early 2000s were also marked by the discovery of several NOX analogs. In 1999, David Lambeth’s team identified the first NOX2 analog, initially named MOX1 (for Mitogen Oxidase 1) and later renamed NOX1 [66]. The presence of NOX1 in the colon suggested a role in host defense and a mitogenic activity. Indeed, in most mammalian species, seven NADPH oxidase (NOX) isoforms have been identified: three closely related isoforms, NOX1, NOX2, and NOX3, which are activated by cytosolic subunits; NOX4, which appears to be constitutively active; and the EF-hand–containing, Ca^2+^-activated isoforms NOX5, DUOX1, and DUOX2. Loss-of-function mutations in NOX genes can result in severe human diseases, with NOX2 deficiency causing primary immunodeficiency (CGD) as previously discussed, DUOX2 deficiency manifesting as congenital hypothyroidism [67] and loss of function mutations in NOX1 in children predisposed to very early-onset inflammatory bowel disease (VEOIBD) [68].

### 2.2. Structure of the Human Flavocytochrome b_558_

Unlike many other membrane proteins of interest, NOX enzymes—particularly neutrophil NADPH oxidase—have long resisted structural characterization. Beyond their membrane nature, the difficulty in producing both the NOX2 and p22*^phox^* membrane protein subunits simultaneously by a recombinant approach and the need for a performant eukaryotic expression system for a complex maturation process including insertion of all cofactors, may explain the delay in their structural characterizations. Thus, the first NOX structures emerged following the identification of NOX homologues in bacteria [61] and the resolution of the structure of a bacterial NOX5 homolog from *Cylindrospermum stagnale* (csNOX), including the heme-binding transmembrane ferric oxidoreductase domain (TM) and the intracellular dehydrogenase domain (DH) responsible for FAD- and NADPH-binding [69]. Indeed, this last work permitted the location of the two hemes orthogonally to the membrane plane. In addition, the csNOX structure allowed the identification of—thanks to a stabilized water molecule in a pocket close to the outer heme—the putative site for oxygen reduction that has been called the oxygen reducing center (ORC). Then, with the rise in cryo-electron microscopy use, the structures of mouse DUOX1 [70] and human DUOX1 [71], more accessible due to their large sizes, were obtained providing the first entire structure of NOX/DUOX family members. These structures permitted the confirmation of the position of the two hemes highlighted in CsNOX, while the ORC—easily accessible from the extracellular side in csNOX5—was completely buried within the DUOX enzymes [69]. Finally, the first structure of the core human NADPH oxidase NOX2 was revealed in 2022 [72]. The TM domain and all three extracellular loops were well resolved, whereas the DH domain was seen at a lower cryo-EM resolution. However, they also confirmed that His101 and H209 (in the TM3 and TM5 of NOX2 respectively) coordinate the inner heme, whereas His115 and His222 (in the TM3 and TM5 of NOX2 respectively) coordinate the outer one (Figure 1A). The importance of hydrophobic residues in between the two hemes, such as Phe215, was proposed to facilitate heme-to-heme electron transfer. In addition, this first structure revealed the long-debated structural architecture of p22*^phox^*, which adopts a four-helix transmembrane fold with two short extracellular loops (Figure 1A). It also highlights—for the first time—interactions between NOX2 and p22*^phox^*. Then shortly after, the human phagocyte NADPH oxidase structure in a resting state was refined by Liu et al. [73]. They showed a hydrophilic tunnel that connects the extracellular environment to the buried oxygen reducing center (ORC) with a radius sufficiently large for the permeation of oxygen and superoxide. This ORC is surrounded by the highly conserved residues R54, H119 and the outer heme. Indeed, a few years ago, in reproducing X91^+^-CGD mutations such as R54G, R54S, and R54M mutations in the NOX2 knock-out PLB-985 cell line model, we demonstrated that R54 was involved in the control of electron flow from FAD to molecular oxygen [74]. The recent NOX2 structures suggest that it is the final electron transfer step to O_2_ that is finally impaired in these CGD mutants. The structure of p22*^phox^* was also specified, notably the polar interaction network formed by side chains of N11 on TM1, E53 on TM2, R90 and H94 on TM3, and Y121 on TM4 of p22*^phox^*. It should be noted that mutations on residues E53, R90, and R94 are responsible for cases of AR22^0^CGD, with the high frequency of mutations at R90 demonstrating a hotspot [75] (Figure 1A). The structure of the FAD-Binding sub-Domain (FBD) of the DH domain was revealed showing P339, F340, T341, and R356 in a pocket for isoalloxazine ring of FAD binding [73] (Figure 1B). Indeed, the absence of FAD incorporation in the T341K X91^+^-CGD PLB-985 cell line was previously shown [76]. In contrast to FAD, the NADPH-binding mode was unresolved, probably due to NADPH’s low affinity for its site in the oxidase’s inactive state. However, they highlighted the interface between NOX2 and p22*^phox^* to structure the heterodimer which involved the TM1 and TM2 of p22*^phox^* and the TM3, TM4 and TM5 of NOX2 [73].

**Figure 2:**
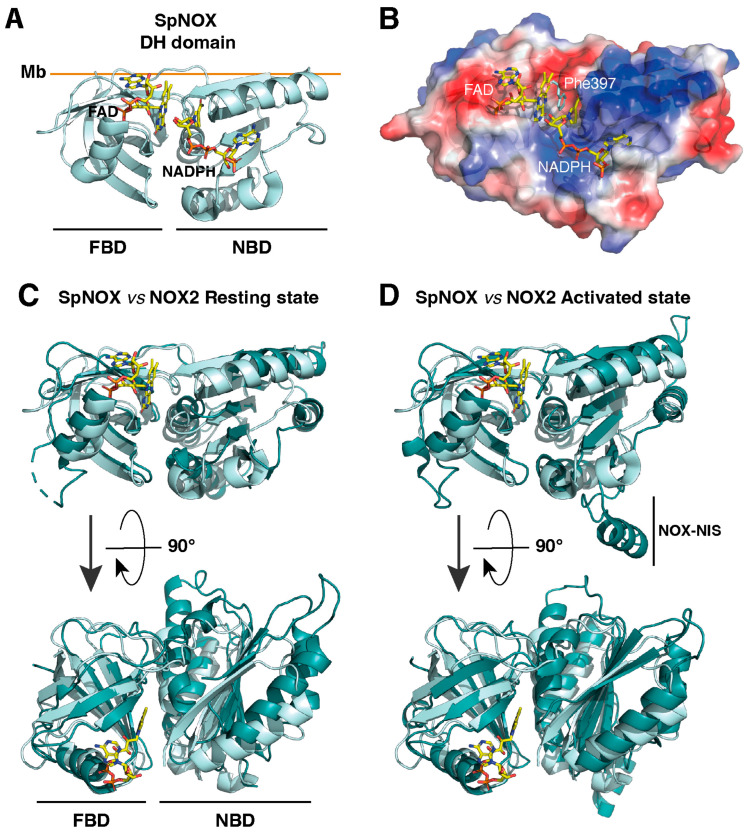
Structural transition from resting to activated state in NOX2 dehydrogenase (DH) domain: (**A**) Structure of the constitutively active dehydrogenase domain of SpNOX as a reference for an active DH domain, i.e., with an efficient hydride transfer from NADPH to FAD. FAD and NADPH are represented highlighting the active site. (**B**) SpNOX DH structure with its surface in an electrostatic mode. Active site cavity with FAD, NADPH and the Phe397 residue, involved in the hydride transfer control, is shown. (**C**) Comparison of the DH domain of active SpNOX versus the inactive resting state of NOX2 DH domain. (**D**) Comparison of the DH domain of active SpNOX versus the activated state of NOX2 DH domain. (**C**,**D**) All DH domains are aligned with respect to the FBD domains of SpNOX in order to highlight the compaction of the NBD in NOX2, upon activation, and its transition towards the SpNOX’s constitutively active DH structure. To note, appearance of the NOX2-NIS structural element in the activated NOX2-DH domain upon complex-formation with p67*^phox^* and Rac (not shown here). NOX2-NIS was disordered, and not visible, in the structure of the resting state of the DH domain. To note for all figures’ clarity, the last 3 C-terminal residues of SpNOX are omitted (residues KFK). Structures used for each DH domain are the following: SpNOX (PDB: 8QQ5 and 8QT7); resting state of NOX2 DH domain (PDB: 8gz3); activated state of NOX2 (PDB: 8wej). Colors: SpNOX (pale cyan), NOX2 (deep teal). FAD and NADPH are represented in sticks using cpk colors, but yellow for carbon atoms. Figures were made using Pymol molecular graphic system v3.1.6.1 and assembled using Abobe Illustrator 30.2.1.

Recently, structural insight on the molecular mechanism of the activation came from structural studies on a bacterial NOX analog, SpNOX [77,78]. This bacterial NOX from *Streptococcus pneumoniae* is constitutively active and thus its structure was believed to represent a reference structure of the activated state. Its main difference with available human NOX2 resting state structure was suggesting the need for a compaction of the DH domain for the activation (Figure 2). These hypotheses were largely confirmed a few months later when the active conformation of the NADPH oxidase complex (comprising NOX2, p22*^phox^*, rac1, p67*^phox^* and part of p47*^phox^*) was unveiled [47]. This NOX2-activated structure permitted the reveal of not only the sequences of interaction between NOX2 and its cytosolic partners, but also the confirmation of the conformational changes in NOX2 induced in the DH domain. Thus, during activation, the DH domain undergoes a combination of two movements: the rotation and the compaction of the domain. Rotation relative to the TM domain brings the FAD closer to the proximal heme, even though the initial distance was already compatible with electron transfer. The compaction of DH, i.e., the closure of the NBD onto the FBD, is particularly important in that it generates the active site allowing NADPH binding and hydride transfer to FAD (see Figure 2 for detailed analysis of the DH compaction upon activation and comparison with the DH domain in Figure 1B,C).

### 2.3. What We Knew About the Synthesis Process of the Flavocytochrome b_558_ Before EROS Discovery

**Figure 3:**
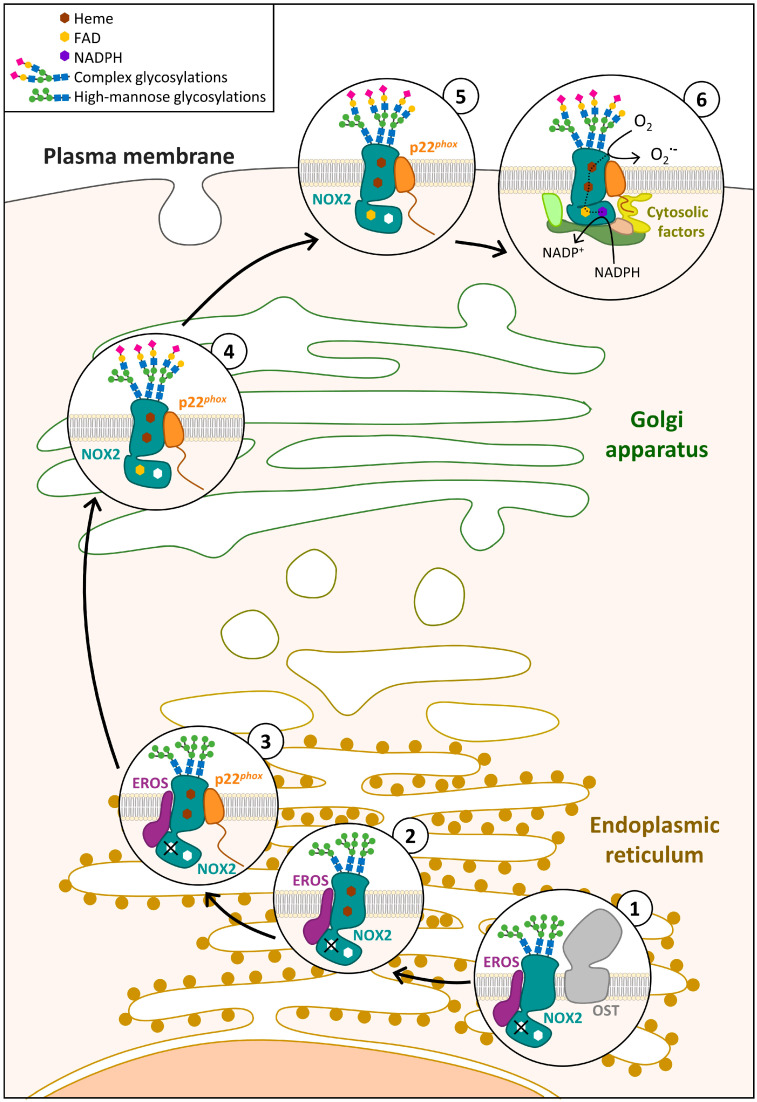
Role of EROS on the different maturation steps during flavocytochrome *b*_558_ synthesis. (1) Co-translational high-mannose glycosylation mediated by the OST complex in the Endoplasmic reticulum—Stabilization of NOX2 gp65*^phox^* precursor by EROS chaperone to prevent its degradation and maintain it in a protected state that does not allow activation. (2) Addition of two hexacoordinated heme cofactors in the transmembrane domain of gp65*^phox^*, still stabilized by EROS chaperone. (3) Heterodimerization with p22*^phox^* to stabilize NOX2 (temporary/transient heterotrimer form with EROS). (4) Release from EROS chaperone induces conformational changes that allow the binding of FAD to its catalytic site in the dehydrogenase domain of NOX2—Change in N-glycosylation profile in the Golgi Apparatus with replacement of high-mannose glycosylation to complex glycosylation in the mature form of NOX2 (gp91*^phox^*). (5) Mature NOX2/p22*^phox^* heterodimers are addressed to the plasma membrane, and the granules or phagosomal membrane. (6) NADPH oxidase activity is triggered thanks to the phosphorylation and subsequent recruitment of cytosolic subunits to the membrane heterodimer. Binding with p67*^phox^* changes NOX2 conformation, which decreases the distance between electron sinks in the DH and TM domains, thus allowing the electron transfer from NADPH to FAD, to the inner then outer hemes and to the final acceptor O_2_ for superoxide production. The X mark/white hexagon represents the FAD binding site blocked by EROS.

The importance of NOX2 and p22*^phox^* co-expression has been evidenced in CGD patients where mutations in one of both genes that disturb the expression of one component results in the decreased production of the other one [31,49]. P22*^phox^* was considered as a “maturation factor” involved in the complete maturation, stabilization and full activity of the heterodimer that it forms with NOX2—but not only as NOX1, NOX3 and NOX4—other members of the NOX/DUOX family need it too for their maturation [79,80,81]. The expression of NOX1-4/p22*^phox^* heterodimers is a complex process that has been largely studied for the phagocyte flavocytochrome *b*_558_. p22*^phox^* is not essential to the initial steps of NOX2 synthesis. First, NOX2 is processed independently of p22*^phox^* and appears first as a p58 KDa precursor co-translationally transformed into a high-mannose glycosylated p65 KDa precursor (gp65*^phox^*) in the early endoplasmic reticulum ER [82]. Heme incorporation in gp65*^phox^* is also independent of the presence of p22*^phox^*, but instead is essential for the heterodimerization with p22*^phox^* in the late ER. The formed heterodimer (known as cytochrome *b*_558_) undergoes complete maturation in the Golgi apparatus, where NOX2 N-glycosylation patterns are remodeled, converting the 65 kDa high-mannose precursor into the mature 91 kDa glycoprotein bearing complex N-glycosylations (gp91*^phox^*/NOX2) (Figure 3) [83,84,85]. In addition, stable heterodimer formation of NOX1-4 isoforms with p22*^phox^* is an essential prerequisite for their localization to specific membrane compartments such as perinuclear vesicles for NOX4, plasma membranes for NOX1-3 and membranes of specific granules for NOX2. However, p22*^phox^* seems to have a specific relationship in its complex with NOX4 compared to complexes with NOX1-3, as some mutations in the membrane-spanning domains of p22*^phox^* (for example the Tyr121His mutation previously found in p22*^phox^* deficient nmf333 mouse) alter NOX1-3 maturation only [86]. In contrast, for NOX1–3, p22*^phox^* plays a crucial role as an anchoring platform for specific sets of cytosolic factors p47*^phox^* [57] or NOXO1 [80,87] making it essential to the activation process, whereas no such process has ever been reported for NOX4.

## 3. EROS Modulates the Biosynthesis of Flavocytochrome *b*_558_ Synthesis

### 3.1. EROS, a Flavocytochrome b_558_ Chaperone Essential for Its Synthesis

Recently, a new partner to the NADPH oxidase complex named EROS as “Essential for Reactive Oxygen Species”, was discovered [13]. In order to highlight new pathways involved in host defense, David Thomas et al. screened knockout mouse lines for susceptibility to infection by inoculating mice generated through the Wellcome Trust Sanger Institute Knockout Mouse Project for Immunity to *Salmonella enterica* serovar Typhimurium challenge [13,88,89]. Mice with a targeted mutation in a previously uncharacterized protein-coding gene located on chromosome 11, named first bc017643, were highly susceptible to *S. Typhimurium* infection. In addition, they demonstrated that this protein was crucial for the generation of the respiratory burst and the neutrophil extracellular traps (NETs) in phagocytes. EROS is also an ortholog of the plant protein Ycf4, necessary for the expression of photosynthetic photo system 1 complex [14]. In addition, several groups described mutations in EROS causing CGD in humans (This will be discussed in detail in Section 4). Therefore, the gene encoding EROS was renamed *CYBC1* for cytochrome B chaperone 1 (for review see [90]).

EROS (formerly known as C17orf62) is a transmembrane protein located in the nuclear envelope [91] and in the endoplasmic reticulum, which is essential for the expression of flavocytochrome *b*_558_ in human phagocytes. EROS co-immunoprecipitated with NOX2 preferentially [12,13]. The direct binding of EROS with NOX2 was ascertained using a yeast 2 hybrid technic and the split luciferase NanoBiT assay [15]. It seems to bind directly to the immature non-glycosylated form of NOX2, p58 KDa and enhance its expression and its stability. This binding prevents NOX2 degradation and promotes the glycosylation process for the maturation and incorporation of hemes, which are essential for the redox activity of NOX2. However, EROS appears to act independently of heme incorporation and the glycosylation process. In addition, the EROS co-transfection experiment with p22*^phox^* in HEK-293 cells did not increase p22*^phox^* expression. Furthermore, size exclusion chromatography provided that EROS can bind heme-bound and non-heme-bound forms of NOX2 and can also be present in a trimeric complex with p22*^phox^* [15]. Thus, EROS can be considered as a stabilizing chaperone acting at the earliest stages of NOX2 maturation (Figure 3).

Overall, thanks to the early studies on NOX2/p22*^phox^* heterodimer formation and maturation (described in detail in Section 2.3), and new insights brought by EROS studies, flavocytochrome *b*_558_ biosynthesis can be summarized as in Figure 3. NOX2 protein is firstly synthesized in the endoplasmic reticulum and co-translationally glycosylated by the OST complex to form gp65*^phox^*, the high-mannose precursor of NOX2 (Figure 3, step 1) [15,82]. During this step, gp65*^phox^* is stabilized by its chaperone EROS, which prevents its degradation [15] and maintains it in a protected state that does not allow spontaneous activation of electron transfer [92]. Maturation of gp65*^phox^* continues in the endoplasmic reticulum by the addition of too hexacoordinated hemes in its transmembrane domain (Figure 3, step 2), still stabilized by EROS. Once hemes are incorporated, NOX2 can interact and bind to its partner p22*^phox^* (Figure 3, step 3) [85] transiently forming a heterotrimer composed of gp65*^phox^* bound to p22*^phox^* on one side, and to EROS on the other side [92]. Gp65*^phox^*/p22*^phox^* heterodimers are released from EROS (which mainly remains in the ER in physiological conditions). Detachment of EROS also triggers conformational changes in the NOX2 DH domain, enabling the incorporation of the FAD in its active site and thus becoming, from that stage, the flavocytochrome *b*_558_. The heterodimer is then processed in the Golgi apparatus where the high-mannose glycosylations are replaced by more complex N-glycosylations to form NOX2 mature form (gp91*^phox^*) (Figure 3, step 4) [82,83,85,93]. Detachment of EROS also triggers conformational changes in the NOX2 dehydrogenase domain, enabling the binding of FAD cofactor inside its catalytic site [47,92]. Mature NOX2/p22*^phox^* complexes are addressed to the membrane (plasmic membrane, granule membrane or phagosomal membrane, depending on cell types and activation triggers) (Figure 3, step 5). Upon cell activation through various mechanisms, which eventually leads to the phosphorylation of both membrane and cytosolic components, the NADPH oxidase complex is assembled with the binding of phosphorylated cytosolic factors (p47*^phox^*, p67*^phox^*, p40*^phox^* and Rac1/2) to NOX2/p22*^phox^* complexes at the membrane site [94,95,96,97,98]. The complex assembly triggers a shift in the dehydrogenase domain of NOX2 that allows the electron transport to occur from the NADPH substrate to the FAD, then to the inner and the outer hemes and finally to the final electron acceptor dioxygen, forming microbicidal superoxide anions in the extracellular space (Figure 3, step 6).

### 3.2. Structure of Human NOX2/EROS Complex

**Figure 4:**
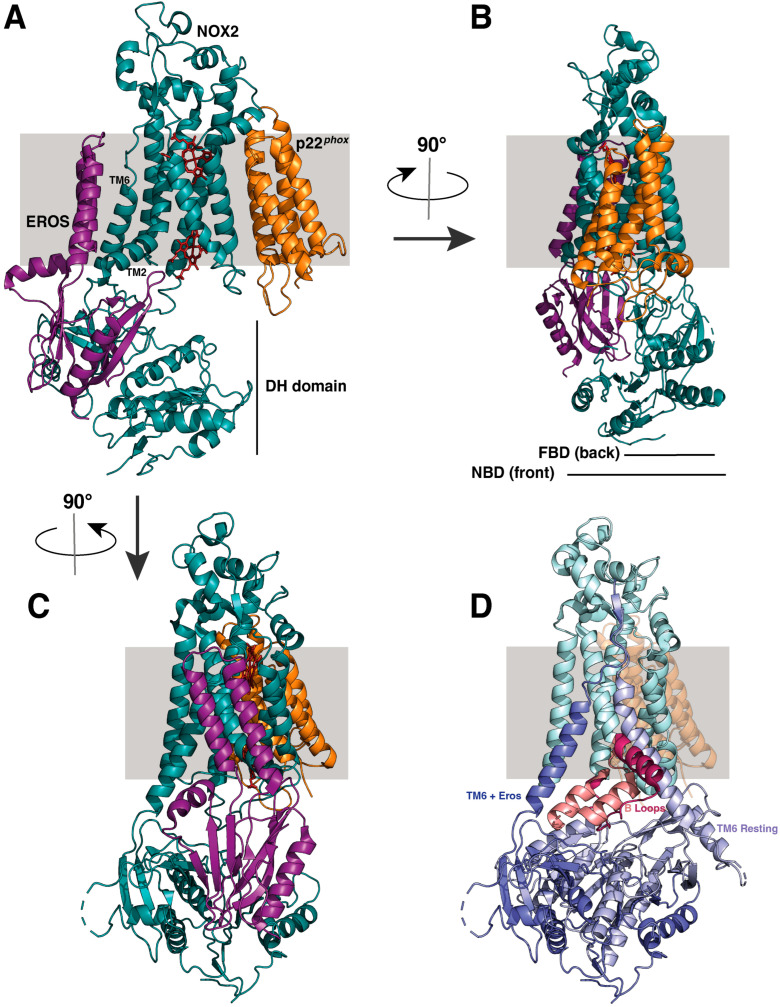
Structure of the EROS/NOX2/p22*^phox^* complex and impact on NOX2 structure. (**A**–**C**) Structures of the EROS/NOX2/p22*^phox^* ternary complex from the lateral side (**A**) or rotated by 90° clockwise presenting p22*^phox^* on the front (**B**) or 90° counterclockwise presenting EROS on the front. (**C**,**D**) Effect of EROS on the conformation of NOX2. To better illustrate its effect on the structure of NOX2, EROS is not represented here. The orientation of the complex is the same here as in (**C**), to aid identification. The NOX2/p22*^phox^* complex is shown with the alternative conformations corresponding to the EROS-bound state and the unbound state (resting state). Whilst p22*^phox^*, at the rear, is unaffected, the structure of NOX2 is profoundly altered. From residue 1 to residue 261, corresponding to the TM domain up to TM5 and the E-loop, the overall structure of NOX2 is preserved (pale cyan), with the exception of the reorientation of the B-loop (colored raspberry and salmon respectively for each state). In contrast, radical conformational changes can be observed at the C-terminal end of NOX2. Thus, when moving from NOX2 complexed with EROS to the resting state structure of NOX2, TM6 shifts from the far left (labeled TM6 + EROS and colored dark blue) to the far right (labeled resting TM and colored light blue) and, consequently, the DH domain, located downstream of TM6, is completely reoriented relative to the TM domain. In all figures, EROS is in deep-purple and p22*^phox^* is in orange. Structures used are the following: EROS/NOX2/p22*^phox^* complex (PDB: 8kei); resting state of resting NOX2/p22*^phox^* (PDB: 8gz3). Figures were made using Pymol molecular graphic system v3.1.6.1 and assembled using Abobe Illustrator 30.2.1.

Very recently, the structure of human EROS in complex with NOX2/p22*^phox^* was unveiled with an overall resolution of 3.56 A [92] (Figure 4A–C). This highlights that EROS and p22*^phox^* interact on opposite sides of NOX2, confirming that EROS and p22*^phox^* do not interact directly [15]. This is also consistent with the fact that EROS has no influence on the expression level of p22*^phox^*. On the contrary, the EROS-NOX2 interface is composed of three parts. The first one is located in the transmembrane part and involves the interaction of the a-helices H1 and H2 of EROS, TM2 and TM6 of NOX2 and intracellular b-strands of EROS. The second contact are polar interactions between the FAD-binding site (FBD) of the DH of NOX2 mainly two residues Q99 and V101 of EROS. In addition, several hydrophobic interactions between the b-strands of EROS and the FBD of NOX2 form a tight binding junction (Figure 4). The third and last interaction portion is with residues of the NADPH-binding sub-Domain (NBD) of the DH domain (the residues G412/P415/G538/E568/F570 of NOX2 and residues R108/R129/A131/T132/G133 of EROS). It is worth noting that missense mutations in the NOX2 residues G412, P415, E568 and F570 are responsible for cases of 91^+^-CGD [76]. Thus, the complex between EROS and NOX2 is completely ineffective for electron transfer. First, the interaction between EROS and NOX2 prevents FAD from accessing its site in the FBD and thus the NOX2/p22*^phox^* complex is not yet a flavocytochrome as long as EROS is associated as a ternary partner. Several major conformational changes can be observed between the heterodimers of NOX2/p22*^phox^* with or without EROS. First, a large conformational change in the B-loop of NOX2 in the continuity of TM2 can be observed (see the two B-loop conformations in pink and raspberry color in Figure 4D). EROS dissociation is also associated with a large reorientation of the TM6 position with respect to the rest of the NOX2 TM domain core (Figure 4D for both alternative conformations). This large movement of TM6 induces a complete rearrangement of the following DH domain with respect to the TM domain. To conclude, EROS sequesters NOX2 in an inactive conformation where the DH domain is lacking the FAD and is not positioned properly with respect to the TM domain and the hemes cofactors (please compare Figure 1A and Figure 4A on one side, as well as Figure 1B,C and Figure 4B on the other side).

Indeed, the EROS/NOX2/p22*^phox^* complex leads one to think that EROS plays not only a chaperone role—protecting NOX2 from denaturation in the early maturation process of NOX2—but could be essential for blocking premature electron transfer within NOX2 to avoid inadequate superoxide production. According to the effect of EROS on the impeachment of FAD and NADPH binding and some preliminary experiments describing the effect of co-overexpression of EROS on the activated NOX2/p22*^phox^*/p47*^phox^*/p67*^phox^* complex in COS cells, EROS seems to play a negative regulatory role of NADPH oxidase activity [92]. However, while the chaperone role is clearly established on the first step of synthesis, this regulatory role on the activity of fully matured complex still needs to be confirmed in more physiological phagocyte models.

Nevertheless, several questions remain unanswered. What mechanism does EROS use to promote NOX2 expression and maturation? What is the proportion of EROS/NOX2 complexes in the plasma membrane and how long does the EROS/NOX2 complex last in the membrane? What is the mechanism regulating the dissociation of EROS from NOX2 to favor FAD incorporation and enabling the electron transfer and where does it occur? These issues have yet to be resolved in order to confirm the role of EROS on the control of the NADPH oxidase activity in phagocytes.

## 4. Does EROS Control ROS-Mediated Cell Production by the NADPH Oxidase Complex?

To answer this question, we chose to assess the impact of EROS mutations on NADPH oxidase activity, as well as the impact of NOX2 mutations located at the interface between the two proteins, which are responsible for rare forms of CGD such as X91^−^-CGD, affecting flavocytochrome *b*_558_ expression and NADPH oxidase activity.

### 4.1. Mutations in EROS/CYBC1 Lead to CGD

Clearly, EROS plays a key role in establishing NADPH oxidase activity in phagocytic cells. Indeed, four types of mutations in EROS in eleven different families are responsible for a novel type of CGD (Table 1). The most frequent mutation was the nonsense mutation c.6C > G leading to the introduction of a stop codon at Tyr2* in EROS and the absence of its expression. The majority of the cases were from Iceland [11], with a high frequency (carried by 1 in 70) suggesting a founder effect. In one patient, no NOX2 expression was detected in monocyte-derived macrophages, whereas a 50% reduction in NOX2 levels was found in his neutrophils. This result is in accordance with the faint NADPH oxidase activity measured in the neutrophils of this patient. This result is also in accordance with previous results in mice where there was minor preservation of the NADPH oxidase activity in response to some stimuli in neutrophils but not in macrophages [13].This discrepency between the EROS deficiency in neutrophils versus macrophages could explain the preponderance of inflammatory episodes in *CYBC1* deficiency patients [11]. Thus, this remains to be confirmed in more than one patient.

Then, Thomas et al. identified an homozygous EROS mutation in a review paper describing a thousand Saudi Arabian families with genetic diseases [12]. One patient presented a missense/splice site mutation (c.127 A>G) and had abnormal NADPH oxidase activity results with both PMA and opsonized zymosan in neutrophils. Absence of EROS expression was validated on anti-CD3-CD28-CD2 expanded T cells from PBMC by Western blot analysis but unfortunatly not in phagocytic cells. However, this mutation in *CYBC1* results in a missense mutation and a splicing defect (p. (Asp43Asn retains intron 3) in EROS—probably deleterious to protein stability. Unfortunatly the expression of NOX2 was not evaluated in the neutrophils and in the expanded T cells of this patient.

A duplication mutation c327dup leading to a missense mutation with a frameshift and the introduction of a stop codon in EROS (p.Val110Cysfs40*) was also described [99]. Due to difficulties in accessing the patient’s blood, Mortimer et al. studied the impact of this mutation on EROS expression reproduced in HEK-293 cells or in NIH3T3 cells by plasmid transfection. Using specific anti-EROS antibodies or with the help of a fusion GFP protein, they demonstrated that the mutation has a drastic effect on EROS expression. In addition, co-expression of EROS with NOX2 in EROS^−/−^ PLB985 cells increases the expression of the 58 kDa precursor of NOX2, mainly confirming the chaperone role of EROS for NOX2. Unfortunatly the impact of this mutation on NADPH oxidase activity in transfected PLB-985 cells was not done.

The last known mutation in *CYBC1* is a deletion c.-8_7delCTCTCGGGATGTACC including the initiation ATG codon [100]. This results in a lack of EROS expression in neutrophils, monocytes and B lymphocytes, leading to a reduction of approximately 50% in NOX2 expression. Thus, after PMA or *E.Coli* stimulation, the NADPH oxidase activity in these cells is consequently reduced too. In contrast to the Tyr2* mutation [11], the absence of EROS expression appears to have the same effect on NOX2 expression and NADPH oxidase activity in neutrophils and monocytes. Unfortunately, no EROS gene mutation results in the synthesis of the corresponding mutated protein, as seen in X91^+^ or in X91^−^-CGDs. It is therefore impossible to study the potential binding defect of mutated EROS with NOX2.

### 4.2. General Clinical Profile of EROS Deficiency in CGD

Up to now less than 20 CGD cases caused by only four different types of mutations in *CYB1C1* gene were discovered. Thus, it is risky to draw a definitive conclusion regarding the clinical profile of these CGD cases. Recently, Bhattarai et al. reported all *CYB1C1* deficiency patient cases in a review [90]. Most of the patients like classical CGD patients suffered from significant infections mostly in lung, skin and soft-tissues, but also from autoimmune/inflammatory manifestations. What is most striking is that they have a higher risk of IBD-like illness than other forms of CGD. However, it is difficult to draw conclusions from such a small cohort. These results need to be confirmed. In particular, are the predominant inflammatory manifestations in these patients due to a more pronounced deficiency of EROS in monocytes/macrophages than in neutrophils, as suggested by Arnadottir et al. [11]? Is EROS involvement in the maturation and expression of NOX1, when mutated, responsible for IBD-like illnesses? [101,102].

### 4.3. Mutations in CYBB That Could Affect EROS/NOX2 Interaction and Flavocytochrome b_558_ Synthesis

In light of the new functional and structural insights recently gained regarding the importance of interactions between EROS and NOX2 for the synthesis of flavocytochrome *b*_558_, we believe it is now possible to re-examine certain missense mutations that had previously remained unexplained. Below, we propose new molecular explanations for certain X91^−^-CGD mutations located in the interaction regions between EROS and NOX2.

#### 4.3.1. The First EROS/NOX2 Interaction Zone Is the Transmembrane Part and Involves the Interaction of the A-Helices H1 and H2 of EROS with TM2 and TM6 of NOX2

**Figure 5:**
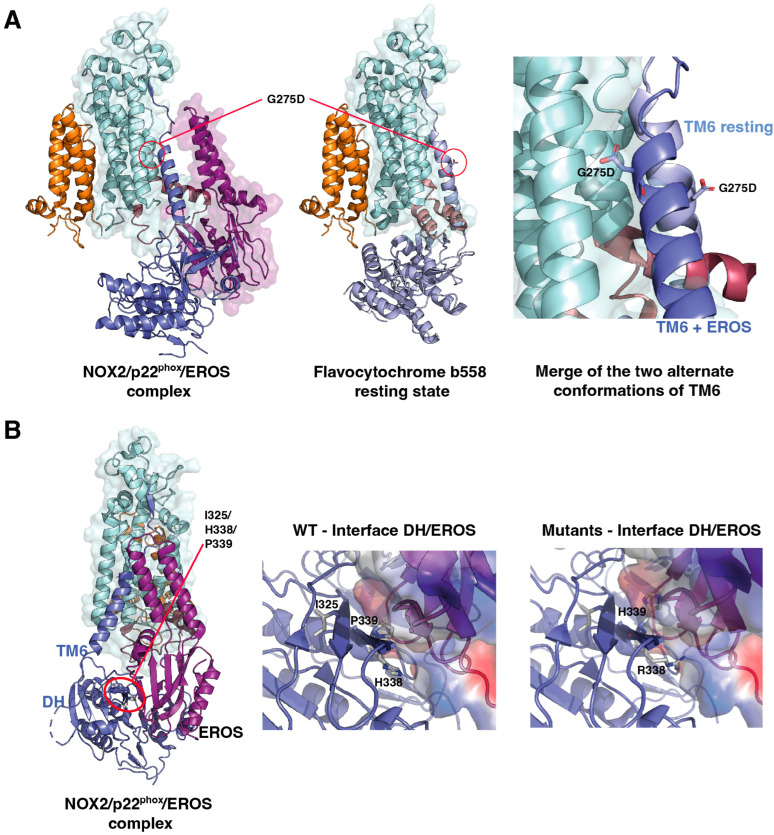
Structural analysis of X91^−^-CGD mutants. (**A**) analysis of the X91^−^-CGD mutation G275D. Left figure, structure of the pre-mature state of the NOX2/p22*^phox^*/EROS complex (contains only the hemes cofactors, not represented here); Middle figure, structure of the mature state of the flavocytochrome *b*_558_ (NOX2/p22*^phox^* heterodimer now containing the FAD cofactor, not represented here). The G275D mutation, located on TM6, whose conformation is strongly modified from one state to another, is highlighted into a red circle. Right figure, zoom on the G275D mutation merging the two alternate conformations (for clarity EROS structure has been omitted on purpose). In all figures, the color code used is the same as described in Figure 4D in order to highlight the conformational changes between different states. The part of the NOX2 structure whose conformation is stable in between both states is in a transparent surface mode. EROS, when present, is in surface mode (**B**) Analysis of the X91^−^-CGD mutations on the cluster of residues I325, H338 and P339. Left figure, the heterotrimeric complex NOX2/p22*^phox^*/EROS where a region corresponding to a cluster of residues, corresponding to X91^−^-CGD mutations, is highlighted in a red circle. On the middle and right side, zoom on the corresponding cluster of residues located on both sides of a small β-sheet involved in the interaction of the NOX2-DH domain with EROS. Middle, Side chain for the wild type residues I325, H338 and P339 are represented as sticks. Right, mutations of residues 338 and 339 to respectively arginine and histidine are represented. In both zooms, the surface of EROS is represented as a transparent electrostatic surface. Structures used are the following; NOX2/p22*^phox^*/EROS complex (PDB: 8kei), resting state of NOX2/p22*^phox^* (PDB: 8gz3). Figures were made using Pymol molecular graphic system v3.1.6.1 and assembled using Abobe Illustrator 30.2.1.

In this region, a G275D mutation of the X91^−^-CGD type has been reported at TM6 of NOX2 [103], but no explanation has been provided as to the impact of this mutation. This residue is located in the middle of TM6, at the heart of the membrane. In the mature, resting state of NOX2, G275 faces the lipid bilayer and, consequently, its mutation to a residue with a longer side chain (such as Asp) should not affect the architecture or stacking of TM6 within the TM domain. Although one might imagine a negative entropic contribution induced by the insertion of such an exposed acidic residue into the hydrophobic bilayer, no structural constraint on the protein’s assembly could be inferred (Figure 5A, middle and right). However, when we now consider the structure of the ternary complex required with EROS during the first stage of synthesis, we can see that TM6 of NOX2 has now been completely rearranged, with position 275 now oriented towards TM1 (Figure 5A, left). In this conformation, a Gly residue at this position is essential, as a clear steric conflict would arise in the event of a G275D substitution. It is reasonable to assume that such a mutation would be detrimental to this conformation of TM6 relative to TM1 and would then compromise the final assembly with EROS. Thus, the chaperone activity could be impeded during the early stages of NOX2 maturation within the ER and could explain the observed expression deficit (see Figure 5A for the different conformations with or without EROS and a close-up view of the two alternative conformations focusing on the G275D mutation).

#### 4.3.2. The Second Part Involves the FAD-Binding sub-Domain Interaction with the β-Sheets of EROS

The β-sheet of NOX2, spanning residues 322 to 341, which plays a key role in FAD binding, forms a major contact interface with the core of the central β-sheets of EROS (see Figure 5, left, within the red circle). Thus, FAD binding and EROS binding are mutually exclusive, as they utilize the same structural elements to interact with NOX2. It is in this β-sheet that a group of X91^−^-CGD mutations was previously identified, involving the three different positions I325, H338 and P339 (see Figure 5B, left and center). The CGD mutations reported at these positions are I325F, H338Y and P339H for the X91^−^-CGD mutants, and H338R for the X91^+^-CGD mutants [76,103,104,105]. When modeling the H338R and P339H mutations (see Figure 5B, right; for graphical reasons, the two mutations are shown together in a single image here), it is evident that the increase in the size of the side chains of the mutated residues will have a steric impact on the interaction interface between NOX2 and EROS, and even an electrostatic impact potentially when considering the H338R mutation *vs.* the electrostatic surface of EROS (Figure 5B, right). Similarly, the H338Y mutation, not modeled here, will likely have an even greater impact on the interface with EROS than the H338R mutation, whose side chain could adapt, reorienting with greater flexibility. This could explain why all these mutants are X91^−^-CGD, with the exception of H338R, which is a X91^+^-CGD mutant. Such mutations could potentially hinder the formation of the NOX2/EROS complex, again leading to a lack of NOX2 stabilization during the initial stages of synthesis.

Finally, the I325F mutation, which is not modeled here, is thought to act via an indirect mechanism, but one similar to the previous one. In a previous work where the existence of such a complex with EROS was not known, I325F mutations has been erroneously attributed to a disrupted interaction with p22*^phox^* instead of EROS [105]. Now, we see that its impact is towards the interaction with EROS. I325 is located on the opposite side of this β-sheet; its mutation to phenylalanine would introduce a bulky aromatic residue into the hydrophobic core of the FBD. To accommodate such a bulky group, it is reasonable to assume that this β-sheet, housing H338 and P339, would be partially pushed outwards from the FBD and would thus exert a steric effect on the interface required for the formation of the complex with EROS.

## 5. Conclusions

In this review, we aimed at synthesizing data from flavocytochrome *b*_558_ biosynthesis, emphasizing both p22*^phox^* and EROS roles on NOX2 stabilization. Using structural information of active and inactive NOX2/p22*^phox^* heterodimers as well as EROS/NOX2/p22*^phox^* heterotrimers, we also inferred the functional implication of EROS and NOX2 mutations in NADPH oxidase activity impairment in Chronic Granulomatous Disease, with a particular emphasis on some formerly unexplained X91^−^-CGD cases.

Most of the early knowledge on flavocytochrome *b*_558_ comes from the study of phagocytes from X-CGD and AR-CGD patients. These first studies led to a better understanding of flavocytochrome *b*_558_ structure, with the discovery of its subunit’s composition, the presence of two hemes with different environments, the importance of FAD cofactors and the outline of its synthesis process. All these findings were then confirmed thanks to recent advances in Crystallography and Cryo-EM analyses, and the flavocytochrome *b*_558_ structure resolution. New data emerging from EROS discovery now perfectly merges with previous studies and filled some of the remaining gaps, for instance in deciphering the first steps of NOX2 synthesis and stabilization. The description of CGD5 mutants also shows the mechanisms by which EROS can impact ROS production in physiological and pathological contexts; thus, in this review we propose new potential explanations for yet unresolved X91^−^-CGD cases, based on the structural analysis of NOX2/EROS complexes.

Although EROS discovery and study have led to a better understanding of flavocytochrome *b*_558_ expression, there remain some unknowns and limits regarding the impact of EROS on flavocytochrome *b*_558_ activation. The low number of published CGD5 cases (only a few natural EROS mutations were described) limits the study of phenotype linked to EROS deficiency. Thus, EROS impact on NADPH oxidase activity is mainly studied in non-physiological cell models, which brings some limits to the conclusions that can be drawn. For instance, the clear EROS inhibitory effect on NADPH oxidase activity was shown in overexpression systems only [92] and might not be as relevant in a physiological context with a lower expression of the chaperone, which is the case in phagocytic cells.

Moreover, the mechanisms by which EROS leads to a stronger expression and maturation of NOX2 should be further studied. EROS localization within the cell should also be precisely addressed in physiological cell models to conclude on the presence or absence of EROS/NOX2 complexes at the plasma membrane or EROS sequestration in the endoplasmic reticulum and nuclear envelope. Overall, this could help contribute to a better understanding of the regulation of NADPH oxidase activity in phagocytes. In particular, future studies should address how EROS chaperone is released (or not) from the cytochrome *b*_558_ during its maturation, and how this release could regulate (or not) the NADPH oxidase complex activation in a physiological context. Another question that remains unanswered—distinct from the EROS contribution this time—is about the triggers and mechanisms for NADPH oxidase complex dissociation after activation, which seems to vary in different phagocyte cell types as they display very different activation/deactivation profiles. Such information would also be of particular interest to better understand the physiology of other NOX isoforms that do not sustain an oxidative burst but rather a lower, but longer and more controlled, NADPH oxidase activity, with diverse cell signalization functions in the homeostasis of the digestive tract, inner ear proper function or renal function. Preliminary results in the HEK293 co-transfection system with a human EROS demonstrated an increased expression of NOX1 and NOX4. In addition, direct binding of EROS to NOX2-4 but not NOX5 or p22*^phox^* was evidenced using a Yeast 2 Hybrid experiment [15]. However, the role of EROS for these different isoforms should be assessed in a more physiological context. Yet, the potential role of other NOXs could also explain the inflammatory phenotypes of CGD5 patients, for example through dysregulation of NOX1 homeostasis for IBD-like symptoms.

## Figures and Tables

**Table 1:** Mutations in *CYBC1* leading to CGD5.

Nucleotide Change	Mutation Type	Amino Acid Change	Protein Expression	Families (Alleles)	References
c.6C>G	Nonsense	p.Tyr2*	No	8 (16)	[11]
c.127 A>G	Missense/splice site	p. (Asp43Asn) retains intron 3 ^1^	No	1 (2)	[12]
c.327dup	Duplication	p.Val110Cysfs40*	No	1 (2)	[99]
c.-8_7delCTCTCG GGATGTACC	Deletion	p.Met1del	No	1 (2)	[100]
c.6C>G	Nonsense	p.Tyr2*	No	1 (2)	[90]

^1^ The variant both disrupts an exonic splice enhancer and creates an exonic splice silencer likely leading a retained intron.

## Data Availability

No new data were created or analyzed in this study. Data sharing is not applicable to this article.

## Abbreviations

The following abbreviations are used in this manuscript:

EROS	Essential for Reactive Oxygen Species
H_2_O_2_	Hydrogen peroxide
OH	hydroxyl radical
HOCl	Perchloric acid
O2^−^	superoxide
ROS	reactive oxygen species
NADPH	Nicotinamide Adenine Dinucleotide Phosphate
PHOX	Phagocytic oxidase
NOX	NADPH oxidase
DUOX	Dual oxidase
DH	Dehydrogenase
FBD	Flavin-Binding sub-Domain
NBD	NADPH-Binding sub-Domain
CGD	chronic granulomatous disease
X-CGD	X-linked Chronic Granulomatous Disease
AR-CGD	Autosomal Recessive Chronic Granulomatous Disease
